# Detecting Endometrial Cancer by Blood Spectroscopy: A Diagnostic Cross-Sectional Study

**DOI:** 10.3390/cancers12051256

**Published:** 2020-05-16

**Authors:** Maria Paraskevaidi, Camilo L. M. Morais, Katherine M. Ashton, Helen F. Stringfellow, Rhona J. McVey, Neil A. J. Ryan, Helena O’Flynn, Vanitha N. Sivalingam, Sarah J. Kitson, Michelle L. MacKintosh, Abigail E. Derbyshire, Cecilia Pow, Olivia Raglan, Kássio M. G. Lima, Maria Kyrgiou, Pierre L. Martin-Hirsch, Francis L. Martin, Emma J. Crosbie

**Affiliations:** 1 School of Pharmacy and Biomedical Sciences, University of Central Lancashire, Preston PR1 2HE, UK; cdlmedeiros-de-morai@uclan.ac.uk (C.L.M.M.); flmartin@uclan.ac.uk (F.L.M.); 2 Department of Metabolism, Institute of Reproductive and Developmental Biology, Digestion and Reproduction & Surgery and Cancer, Imperial College London, London W12 0HS, UK; o.raglan@imperial.ac.uk (O.R.); m.kyrgiou@imperial.ac.uk (M.K.); 3 Pathology Department, Lancashire Teaching Hospitals NHS Foundation, Preston PR2 9HT, UK; katherine.ashton@lthtr.nhs.uk (K.M.A.); helen.stringfellow@lthtr.nhs.uk (H.F.S.); 4 Pathology Department, Manchester University Hospitals NHS Foundation Trust, Manchester M13 9WL, UK; rhona.mcvey@mft.nhs.uk; 5 Division of Cancer Sciences, University of Manchester, Manchester M13 9WL, UK; neilryan@nhs.net (N.A.J.R.); helena.oflynn@manchester.ac.uk (H.O.F.); vanitha.sivalingam@manchester.ac.uk (V.N.S.); sarah.kitson@manchester.ac.uk (S.J.K.), ceci.pow@live.co.uk (C.P.); emma.crosbie@manchester.ac.uk (E.J.C.); 6 Department of Obstetrics and Gynaecology, Manchester University Hospitals NHS Foundation Trust, Manchester M13 9WL, UK; michelle.mackintosh@mft.nhs.uk (M.L.M.); abiderbyshire@doctors.net.uk (A.E.D.); 7 Department of Chemistry, Biological Chemistry and Chemometrics, Federal University of Rio Grande do Norte, Natal 59072-970, Brazil; kassiolima@gmail.com; 8 West London Gynaecological Cancer Centre, Queen Charlotte’s & Chelsea–Hammersmith Hospital, Imperial Healthcare NHS Trust, London W12 0HS, UK; 9 Department of Obstetrics and Gynaecology, Lancashire Teaching Hospitals NHS Foundation, Preston PR2 9HT, UK; martin.hirsch@mac.com

**Keywords:** blood diagnostics, endometrial cancer, screening, spectroscopy

## Abstract

Endometrial cancer is the sixth most common cancer in women, with a rising incidence worldwide. Current approaches for the diagnosis and screening of endometrial cancer are invasive, expensive or of moderate diagnostic accuracy, limiting their clinical utility. There is a need for cost-effective and minimally invasive approaches to facilitate the early detection and timely management of endometrial cancer. We analysed blood plasma samples in a cross-sectional diagnostic accuracy study of women with endometrial cancer (*n* = 342), its precursor lesion atypical hyperplasia (*n* = 68) and healthy controls (*n* = 242, total *n* = 652) using attenuated total reflection-Fourier transform infrared (ATR-FTIR) spectroscopy and machine learning algorithms. We show that blood-based infrared spectroscopy has the potential to detect endometrial cancer with 87% sensitivity and 78% specificity. Its accuracy is highest for Type I endometrial cancer, the most common subtype, and for atypical hyperplasia, with sensitivities of 91% and 100%, and specificities of 81% and 88%, respectively. Our large-cohort study shows that a simple blood test could enable the early detection of endometrial cancer of all stages in symptomatic women and provide the basis of a screening tool in high-risk groups. Such a test has the potential not only to differentially diagnose endometrial cancer but also to detect its precursor lesion atypical hyperplasia—the early recognition of which may allow fertility sparing management and cancer prevention.

## 1. Introduction

There has been a steady rise in the incidence of endometrial cancer in the Western world [1], with the UK reporting a 56% rise over the last two decades [2]. In 2018, there were 380,000 new cases of endometrial cancer worldwide, rendering it the sixth most common cancer in women [3]. The rising incidence of endometrial cancer has been attributed to the emerging obesity epidemic [4], fewer hysterectomies for benign indications and the ageing population [5].

Endometrial cancer typically presents with postmenopausal bleeding, yet only 5–10% of symptomatic women have underlying cancer [6]. Symptoms are usually investigated by measuring endometrial thickness using transvaginal ultrasound scan (TVS) in the first instance [7]. This is an intimate procedure, costly, operator dependent and limited by the scarcity of trained ultrasonographers. Different endometrial thickness cut-offs also affect its clinical utility [5,8]. Women with a thickened endometrium undergo invasive sampling for histological assessment, either in the outpatient setting or under general analgesia [5]. This is an invasive test that is poorly tolerated by some and yields insufficient tissue for diagnosis in up to 22% of cases [9].

Whilst the majority of women are diagnosed with stage 1 disease in the UK, 30% are diagnosed with advanced disease and have a poor prognosis [2]. Earlier recognition of disease by screening might enable stage shift and improve patient outcomes [10]. In the United Kingdom Collaborative Trial of Ovarian Cancer Screening (UKCTOCS) cohort, screening average-risk women for endometrial cancer by TVS had inferior diagnostic accuracy compared to symptomatic women for standard test thresholds [8].

Blood biomarkers have the potential to provide an inexpensive screening/diagnostic tool for endometrial cancer. Such a tool could be used on its own or as a means of triaging women for further tests and may be more acceptable to women than current investigations because it does not require intimate examination. The search for circulating biomarkers to facilitate the early detection of cancer has gained great momentum over the last decade [11,12,13].

Vibrational spectroscopy explores the vibrations induced to molecules’ chemical bonds after exposure to electromagnetic radiation, thus providing valuable information about a biological sample and its constituents [14]. The two main vibrational spectroscopic techniques, namely infrared and Raman spectroscopy, are sensitive to different types of vibrations and therefore provide complementary spectral information. One of the major advantages of spectroscopy is that it is not limited to investigating one biomolecule at a time but instead simultaneously examines the full range of molecules present within a sample, including proteins, lipids and nucleic acids. Previous research has demonstrated the promise of spectroscopy as a means of diagnosing and classifying Alzheimer’s disease, AIDS, ovarian cancer and brain cancer, amongst others [15,16,17,18]. The easy accessibility and information-rich nature of blood samples hold promise not only for the diagnosis of disease but also for the assessment of prognosis or response to treatment.

In this large cross-sectional diagnostic study, we used blood-based infrared spectroscopy to differentiate healthy individuals from women with endometrial cancer and its precursor lesion, atypical hyperplasia. We included women with the full range of histological subtypes, grades and stages of cancer. We sought to identify the spectral regions that were responsible for the differentiation between the various pathologies. We also determined whether blood-based spectroscopy could detect cancer at its earliest stage and even its precursor lesion, atypical hyperplasia, which may prevent the development of endometrial cancer [19].

## 2. Results

### 2.1. Participant Demographics

Our study population comprised 652 women, with 242 healthy controls, 342 women with endometrial cancer (Type I: 258; Type II: 64; Mixed: 20) and 68 with atypical hyperplasia (Table 1). The mean age of the healthy controls, women with Type I cancers, Type II cancers and atypical hyperplasia was 52 years (standard deviation SD 11), 63 years (SD 13), 69 years (SD 12) and 52 years (SD 15), respectively. Information for the stage of the disease was recorded: 68 patients had atypical hyperplasia (Stage 0), 192 patients had FIGO 2009 Stage IA cancer, 59 patients Stage IB, 3 patients were staged as IA/1B (indeterminate), 29 patients were Stage II, 52 were Stage III and 6 were Stage IV; one patient’s stage was missing from our records. The healthy controls were significantly younger than the cancer patients (52 years vs. 63–69 years (for Type I and Type II cancers respectively), *p* < 0.0001), but not those with atypical hyperplasia (52 years vs. 52 years, *p* = 1.000). After taking all the groups into account, age was not found to be statistically independent (*p* = 0.268). BMI was not statistically significant in the control vs. Type I (*p* = 0.220) and control vs. Type II comparisons (*p* = 0.241) as well as mixed cancers (*p* = 0.220) and atypical hyperplasia (*p* = 0.220). Overall, BMI showed no statistical independence between the groups (*p* = 0.412). Self-reported diabetes (*p* = 0.268), blood pressure (*p* = 0.268) and fasting status (*p* = 0.345) did not show statistical independence when considering all classes.

### 2.2. Endometrial Cancer and Pre-Cancer Diagnosis

**Figure 1:**
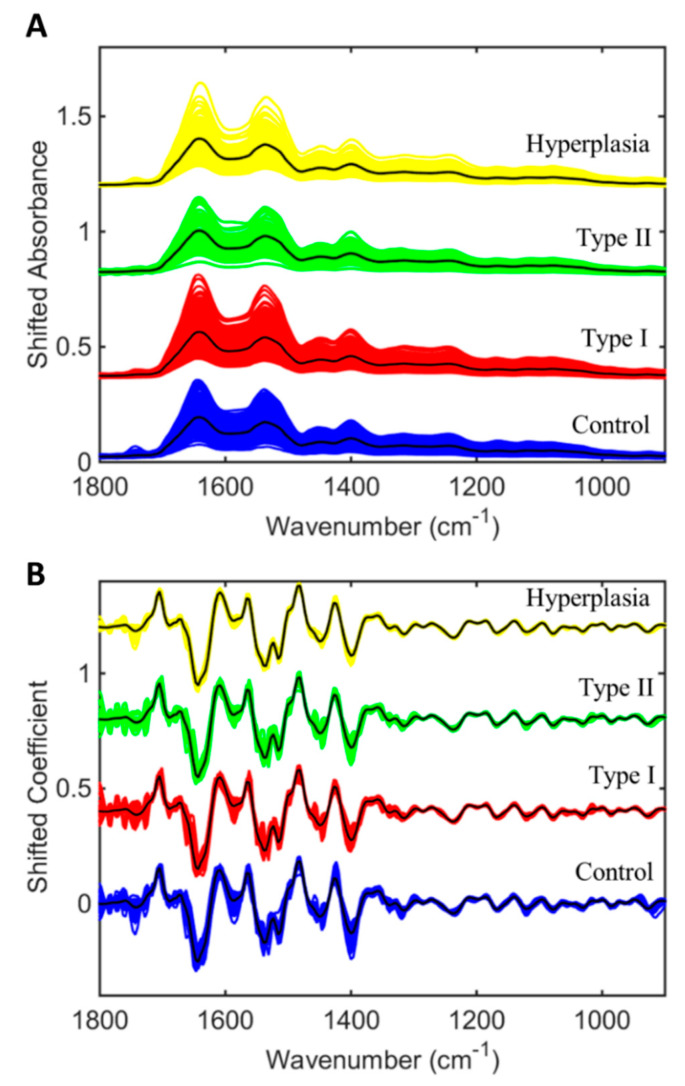
Infrared spectral data for the healthy controls (*n* = 242), Type I cancers (*n* = 258), Type II cancers (*n* = 64) and atypical endometrial hyperplasia (*n* = 68) at the fingerprint region (1800–900 cm^−1^). (**A**) Raw infrared spectra for the different classes. (**B**) Pre-processed spectra after 2nd Savitzky–Golay (SG) derivative (window of 5 points, 2nd-order polynomial fitting) and vector normalization. Coloured lines denote all spectra, while black line shows the average spectrum.

**Figure 2:**
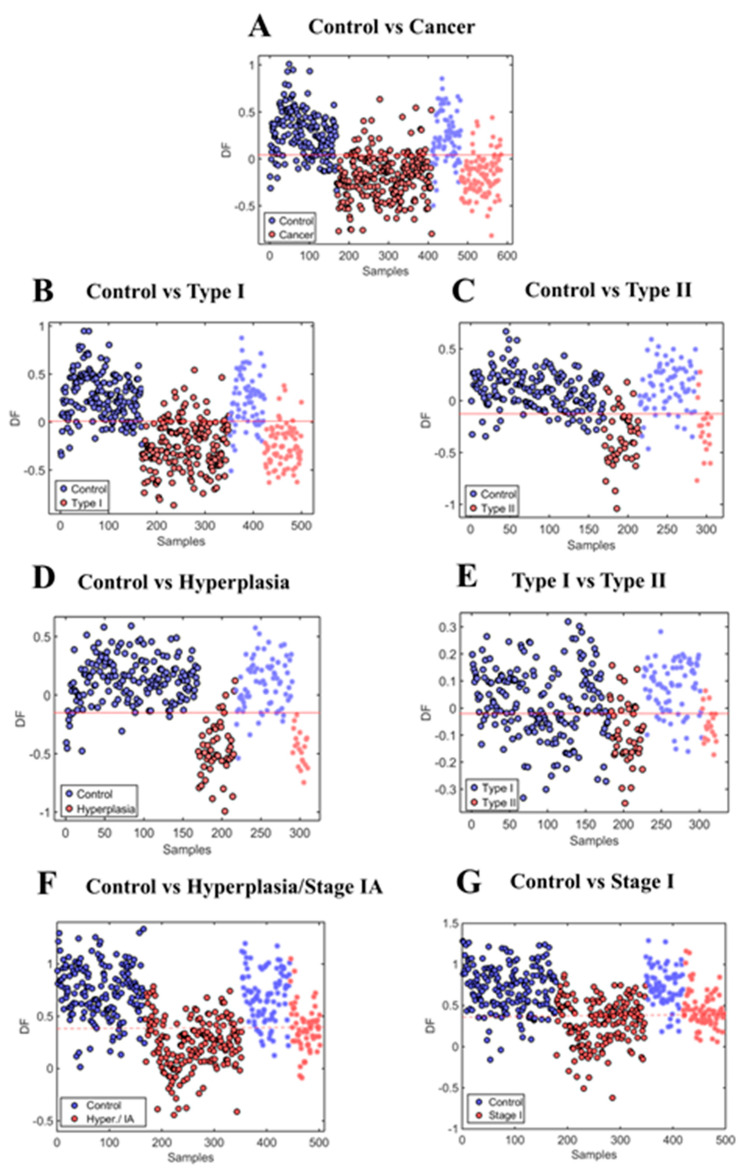
Discriminant function (DF) graphs showing the differences and similarities between the different classes after supervised partial least squared discriminant analysis (PLS-DA). (**A**) Control (*n* = 242) vs. cancer (*n* = 342; including Type I (*n* = 258), Type II (*n* = 64) and mixed (*n* = 20)). (**B**) Control (*n* = 242) vs. Type I cancers (*n* = 258). (**C**) Control (*n* = 242) vs. Type II (*n*=64) cancers. (**D**) Control (*n* = 242) vs. hyperplasia (*n* = 68). (**E**) Type I (*n* = 258) vs. Type II cancers (*n* = 64). (**F**) Control (*n* = 242) vs. hyperplasia/Stage IA (*n* = 260). (**G**) Control (*n* = 242) vs. Stage I (*n* = 254). o: training samples; *: test samples.

After blood analysis, all raw spectroscopic data (Figure 1A and Figure S1) were pre-processed to correct for non-biological interference that could lead to misinterpretation of the results. The pre-processed spectra (Figure 1B) were then used for multivariate analysis and class differentiation according to the final histopathological diagnosis. Five sequential spectra were collected from each patient, resulting in a total number of 3260 spectra. Discriminant function (DF) graphs were generated to show the differences and similarities between the different classes (Figure 2 and Figure S5). Figure 2 shows both the samples used in the training dataset and the ones used in the test dataset for each comparison. For our main analysis, we compared controls with all cancer cases (Figure 2A), controls with Type I cancers (Figure 2B), controls with Type II cancers (Figure 2C), controls with hyperplasia (Figure 2D), Type I and Type II cancers (Figure 2E), controls with hyperplasia/Stage IA cancers (Figure 2F), and controls with Stage I cancers (Figure 2G). We also performed additional subgroup comparisons to compare hyperplasia against Type I cancers (Figure S5A,B), hyperplasia against Type II cancers (Figure S5C,D), and hyperplasia against cancer (Figure S5E,F). The mean pre-processed spectra for all the different comparisons are given in Figure S7.

**Figure 3:**
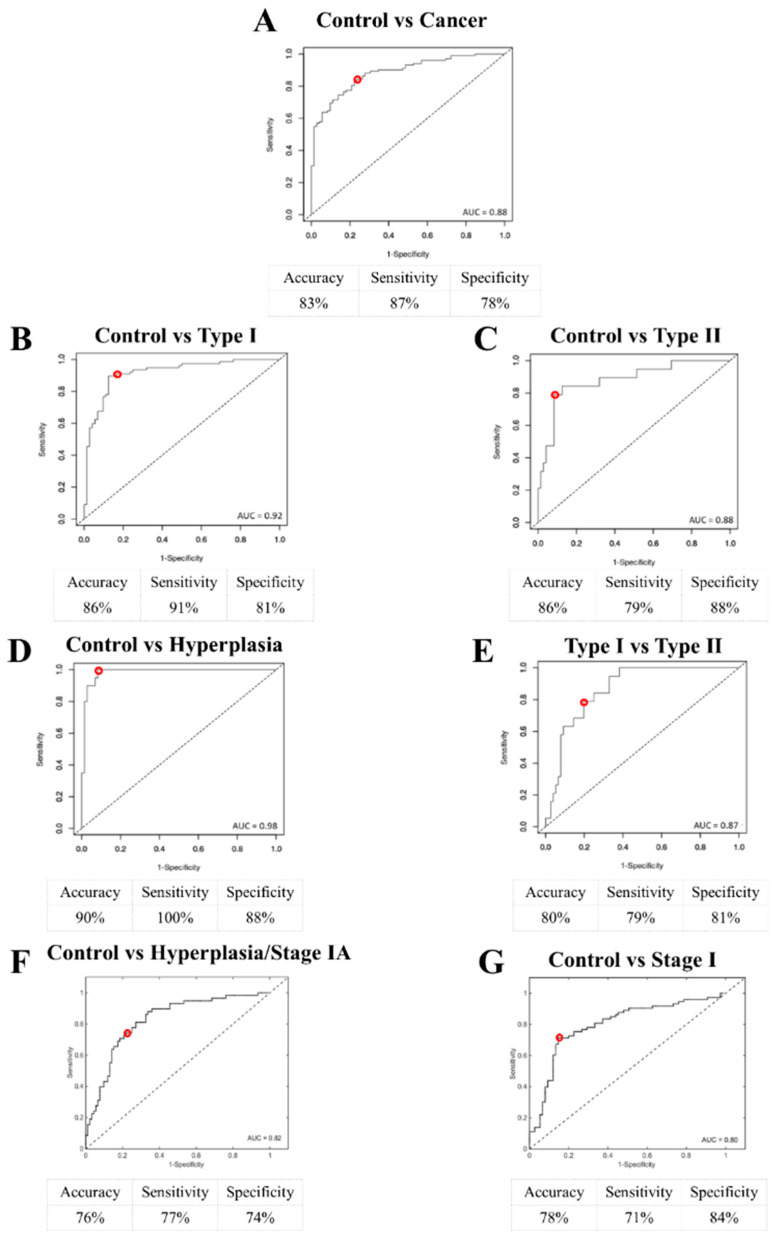
Receiver operating characteristic (ROC) curves along with overall accuracies, sensitivities, specificities and area under the curve (AUC) values after supervised partial least squares discriminant analysis (PLS-DA). (**A**) Control (*n* = 242) vs. cancer (*n* = 342; including Type I (*n* = 258), Type II (*n* = 64) and mixed (*n* = 20)). (**B**) Control (*n* = 242) vs. Type I cancers (*n* = 258). (**C**) Control (*n* = 242) vs. Type II (*n* = 64) cancers. (**D**) Control (*n* = 242) vs. hyperplasia (*n* = 68). (**E**) Type I (*n* = 258) vs. Type II cancers (*n* = 64). (**F**) Control (*n* = 242) vs. hyperplasia/Stage IA cancer (*n* = 260). (**G**) Control (*n* = 242) vs. Stage I cancer (*n* = 254). The red circle denotes the cut-off point for the optimal compromise between sensitivity and specificity.

Receiver operating characteristic (ROC) curves were used to calculate the area under the curve (AUC) and find a compromise between sensitivity and specificity [20]. Comparing healthy controls with cancer generated an AUC of 88%, 87% sensitivity and 78% specificity (overall accuracy of 83%) (Figure 3A). When healthy controls were compared with Type I cancers alone, the AUC increased to 92%, sensitivity to 91% and specificity to 81% (overall accuracy of 86%) (Figure 3B). Comparing controls to Type II endometrial cancers resulted in an 88% AUC, 79% sensitivity and 88% specificity (overall accuracy of 86%) (Figure 3C). After comparison of the controls with the hyperplasia cases, AUC was 98%, sensitivity 100% and specificity 88% (overall accuracy of 90%) (Figure 3D). Comparison of the two subtypes of endometrial cancer, Type I and Type II, gave an AUC of 87%, sensitivity 79% and specificity 81% (overall accuracy of 80%) (Figure 3E). Comparing controls to hyperplasia/Stage IA cancers gave an AUC of 82%, 77% sensitivity and 74% specificity (overall accuracy of 76%). Finally, after the comparison between controls and Stage I cancers, the AUC was 80%, sensitivity 71% and specificity 84% (overall accuracy of 78%). Comparing hyperplasia against Type I cancer generated a 71% AUC with 60% sensitivity and 77% specificity (overall accuracy of 73%) (Figure S6); hyperplasia against Type II cancer achieved 95% AUC, 90% sensitivity and 84% specificity (overall accuracy 87%); and hyperplasia against cancer achieved 80% sensitivity and 71% specificity (overall accuracy of 76%) (Figure S6).

### 2.3. Panel of Potential Diagnostic Spectral Markers

**Figure 4:**
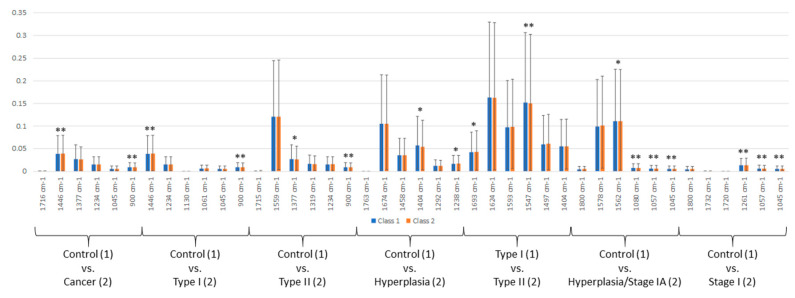
The six most discriminatory peaks for each subgroup analysis detected after partial least squared discriminant analysis (PLS-DA). The differences in the absorbance levels are given as the mean ± standard deviation and were calculated after automatic weighted least squares baseline correction and vector normalization. * *p* < 0.05; ** *p* < 0.005.

The spectral peaks responsible for the segregation between the different classes were identified and their absorbance levels were recorded and compared to allow quantitative assessment (Figure 4, Figure S7, Table S1). The discriminatory peaks between healthy controls and cancers were 1716 cm^−1^ (*p* = 0.183), 1446 cm^−1^ (*p* = 0.003), 1377 cm^−1^ (*p* = 0.060), 1234 cm^−1^ (*p* = 0.05), 1045 cm^−1^ (*p* = 0.415) and 900 cm^−1^ (*p* < 0.0001). The two peaks that were found to be significantly different were tentatively assigned to chemical bonds and potential biological molecules: a peak at 1446 cm^−1^, indicative of –CH_2_ bending vibrations (lipids), was increased in cancer while a peak at 900 cm^−1^, indicative of C-O or C-C stretching (carbohydrates) or fatty acids, was decreased (Figure 4) [21,22]. The peaks that were found to differentiate controls from Type I cancers were 1446 cm^−1^ (*p* = 0.002), 1234 cm^−1^ (*p* = 0.123), 1130 cm^−1^ (*p* = 0.349), 1061 cm^−1^ (*p* = 0.231), 1045 cm^−1^ (*p* = 0.321) and 900 cm^−1^ (*p* < 0.0001). As before, the same peaks were statistically significant, with 1446 cm^−1^ increased and 900 cm^−1^ decreased in cancer cases (Figure 4). After comparison of controls and Type II cancers, the following peaks were detected: 1715 cm^−1^ (*p* = 0.92), 1559 cm^−1^ (*p* = 0.062), 1377 cm^−1^ (*p* = 0.043), 1319 cm^−1^ (*p* = 0.133), 1234 cm^−1^ (*p* = 0.158) and 900 cm^−1^ (*p* < 0.0001); both peaks at 1377 cm^−1^(stretching C-N cytosine, guanine) and 900 cm^−1^ (C-O or C-C stretching (carbohydrates) or fatty acids) were decreased in Type II cases (Figure 4). Peaks at 1763 cm^−1^ (*p* = 0.843), 1674 cm^−1^ (*p* = 0.257), 1458 cm^−1^ (*p* = 0.885), 1404 cm^−1^ (*p* = 0.041), 1292 cm^−1^ (*p* = 0.823) and 1238 cm^−1^ (*p* = 0.017) were responsible for the differentiation between controls and women with hyperplasia. The absorbance level at peak 1404 cm^−1^ (symmetric bending of -CH_3_ in proteins) was decreased, while that at 1238 cm^−1^ (asymmetric PO_2_^−^ stretching, collagen and nucleic acids) was increased in hyperplasia (Figure 4). After comparing the different subtypes of cancer, the six differential peaks were 1693 cm^−1^ (*p* = 0.006), 1624 cm^−1^ (*p* = 0.263), 1593 cm^−1^ (*p* = 0.115), 1547 cm^−1^ (*p* < 0.0001), 1497 cm^−1^ (*p* = 0.072) and 1404 cm^−1^ (*p* = 0.494), with 1693 cm^−1^ (Amide I of proteins) being elevated in Type II and 1547 cm^−1^ (Amide II of proteins) being decreased in Type II (Figure 4). Comparison between controls and hyperplasia/Stage IA cancers showed the following peaks as the most discriminatory: 1800 cm^−1^ (*p* = 0.356), 1578 cm^−1^ (*p* = 0.123), 1562 cm^−1^ (*p* = 0.041), 1080 cm^−1^ (*p* < 0.001), 1057 cm^−1^ (*p* < 0.0001) and 1045 cm^−1^ (*p* < 0.0001). All four absorbance peaks that showed statistical significance were decreased in the hyperplasia/Stage IA group: 1562 cm^−1^ (Amide II), 1080 cm^−1^ (symmetric PO_2_^−^ stretching), 1057 cm^−1^ (stretching C-O deoxyribose) and 1045 cm^−1^ (carbohydrates). Finally, after comparing controls and Stage I cancers, the detected peaks were: 1800 cm^−1^ (*p* = 0.478), 1732 cm^−1^ (*p* = 0.665), 1720 cm^−1^ (*p* = 0.579), 1261 cm^−1^ (*p* < 0.0001), 1057 cm^−1^ (*p* = 0.001) and 1045 cm^−1^ (*p* = 0.001), with 1261 cm^−1^ (asymmetric PO_2_^−^ stretching) being increased in Stage I cancers and 1057 cm^−1^ (stretching C-O deoxyribose), 1045 cm^−1^ (carbohydrates) being decreased.

### 2.4. Consideration of Potential Confounding Factors

**Figure 5:**
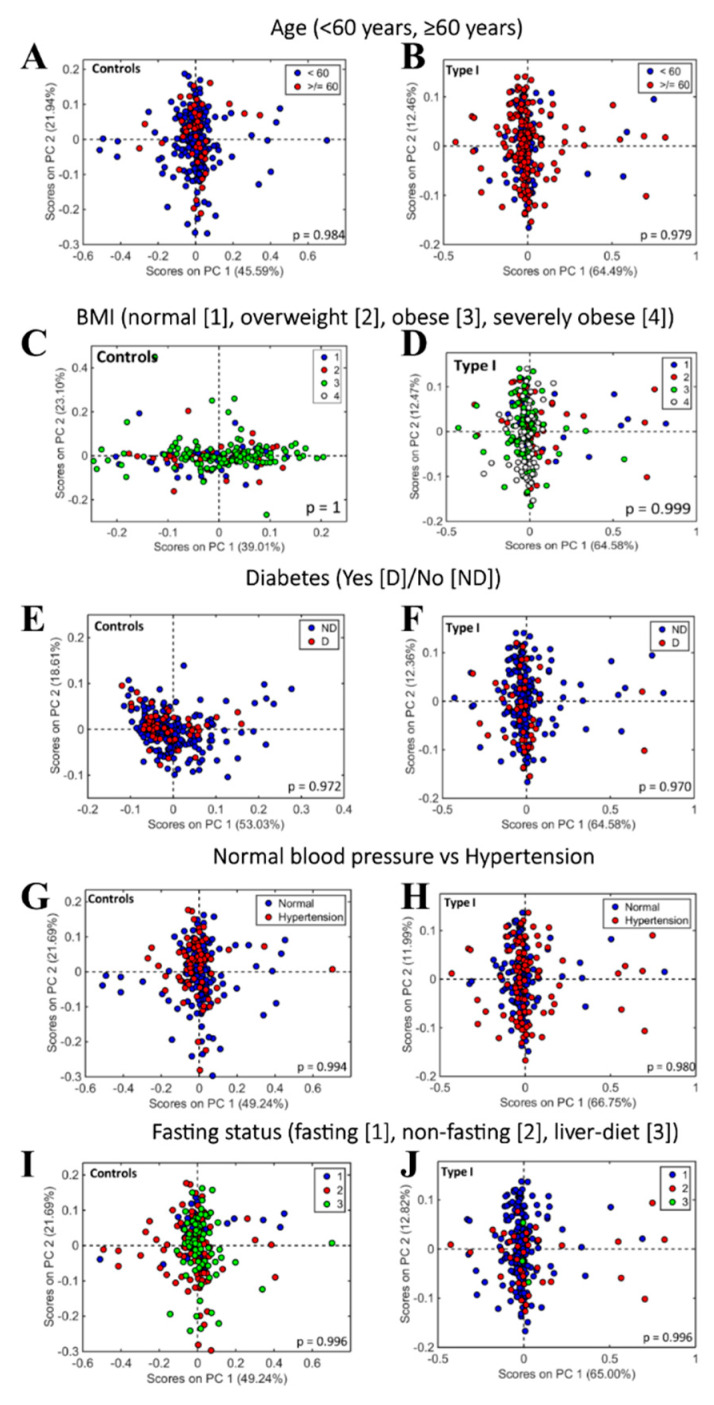
Score plots generated after unsupervised principal component analysis (PCA) to visualize differences and similarities according to confounding factors. (**A,B**) Score plots according to age (<60 years; ≥60 years) for controls (**A**) and Type I cancers (**B**). (**C**,**D**) Score plots according to BMI (normal: BMI = 18.5–24.9; overweight: BMI = 25–29.9; obese: BMI = 30–39.9; severely obese: BMI > 40) for controls (**C**) and Type I cancers (**D**). (**E**,**F**) Score plots according to diabetes (diabetic; non-diabetic) for controls (**E**) and Type I cancers (**F**). (**G**,**H**) Score plots according to fasting status (fasting; non-fasting; liver diet) for controls (**G**) and Type I cancers (**H**). (**I**,**J**) Score plots according to blood pressure (normal; hypertension) for controls (**I**) and Type I cancers (**J**).

In order to confirm that the diagnostic performance generated from the subgroup comparisons was attributable to the presence/absence of disease, we performed further analyses to take into account potential confounding factors that could affect the results (Figure 5). These conditions were tested on the healthy controls and the Type I cancers because of the greater number of participants in these groups. An unsupervised exploratory analysis by means of principal component analysis (PCA) was performed to identify differences and similarities between the groups. The PCA scores plot did not show any segregation pattern among the confounding factor comparisons, indicating that age, BMI, diabetes, fasting status and blood pressure did not affect the spectral response within each class of sample. In addition, no statistical significance was found at a 95% confidence level for spectral differences in age (<60 vs. ≥60 years) for either controls (*p* = 0.984) or Type I cancers (*p* = 0.979) (Figure 5A,B); BMI (normal vs. overweight vs. obese vs. severely obese) for controls (*p* = 1) or Type I cases (*p* = 0.999) (Figure 5C,D); diabetes (diabetic vs. non-diabetic) for controls (*p* = 0.972) or Type I (*p* = 0.97) (Figure 5E,F); blood pressure (normotension vs. hypertension) for controls (*p* = 0.994) or Type I cancers (*p* = 0.980) (Figure 5G,H); and fasting status (fasting vs. non-fasting vs. liver diet) for controls (*p* = 0.996) or Type I (*p* = 0.996) (Figure 5I,J) based on a MANOVA test applied to the spectral wave numbers. There was no difference in the quality of spectra and the diagnostic performance of blood spectroscopy on samples taken from gynaecology clinics in Manchester or Lancashire (Figure S2).

## 3. Discussion

We evaluated whether infrared spectroscopy could detect endometrial cancer and its precursor lesion, atypical hyperplasia, in blood samples. In this large diagnostic test accuracy study, we achieved sensitivities of 71–100% and specificities of 81–88% for detection of disease, highlighting the potential of spectroscopy as an inexpensive diagnostic and/or screening tool.

We examined the spectroscopic profiles to identify the peaks responsible for distinguishing between cases and controls and found six discriminatory features, which could serve as a panel of spectral markers indicative of disease. We found that cancer cases were associated with increased lipid-related (peak at 1446 cm^−1^) and decreased carbohydrate- and fatty acid-related regions (peaks at 1377 and 900 cm^−1^). The elevated expression of the lipid species has been described before [23,24,25], but the lower carbohydrate- and fatty acid-related peaks is a novel finding, perhaps originating from overlapping absorptions of other biomolecules. Two spectral markers were identified as statistically different in atypical hyperplasia compared to the cancer cases: a decreased peak at 1404 cm^−1^, potentially corresponding to proteins, and an increased peak at 1238 cm^−1^, corresponding to collagen and nucleic acids. Both peaks related to proteins (Amide I at 1693 cm^−1^ and Amide II at 1547 cm^−1^) showed statistically significant increases in Type II when compared to Type I cancers. After comparing controls with early stage cases (hyperplasia/Stage IA or Stage I), the latter revealed decreased levels of absorbance in regions representing proteins (peak at 1562 cm^−1^), nucleic acids (peaks at 1080 and 1057 cm^−1^) and carbohydrates (peak at 1045 cm^−1^); only peak 1261 cm^−1^ (indicative of asymmetric PO_2_^−^) was found to be increased in Stage I cancers.

To investigate the effect of confounding factors within each class and to establish that the differences between classes were attributable to disease per se, we took into consideration patient characteristics associated with an increased risk of endometrial cancer. Age [5], obesity [26], diabetes [27] and hypertension [28] are all established risk factors for the disease [29,30]; we also controlled for fasting status, as this was expected to affect spectroscopic signal. Nevertheless, after exploratory analysis, these factors alone were not sufficient to generate statistical significance within groups, indicating that the diagnostic performance of spectroscopy in this study related to the presence or absence of disease.

We found that the sensitivity and specificity of spectroscopy was sufficiently high to have clinical utility, justifying its potential incorporation into future clinical practice diagnostic algorithms. Since sensitivity was generally higher than specificity, spectroscopy could be most valuable as a first-line diagnostic test that safely reassures healthy women and identifies women at highest risk of disease for invasive diagnostic procedures. Such a strategy should be tested in a prospective diagnostic accuracy study before its assimilation in clinical practice. For comparison, in a meta-analysis of 90 studies assessing the diagnostic accuracy of TVS at different endometrial thickness thresholds [31], the authors demonstrated a sensitivity of 94.8% (95% CI 86.1–98.2%) and specificity of 46.7% (95% CI 38.3–55.2%) for 4 mm and 90.3% (95% CI 80.0–95.5%) and 54.0% (95% CI 46.7–61.2%) for 5 mm, respectively. Our spectroscopic blood test achieved comparable sensitivity of 87% and superior specificity of 78% for diagnosing cancer, which highlights its clinical value.

Effective cancer management is dependent on early detection, when treatment is most likely to be curative; this has led to the search for novel imaging and blood biomarkers [13,32]. A ‘cancer blood test’ was ranked as the second most important research priority from a total of 1362 suggestions from 554 patients, the public and health care providers in our recently completed James Lind Alliance Priority Setting Partnership for Detecting Cancer Early [33]. Currently, there is no blood biomarker with sufficient diagnostic accuracy to benefit patient management in endometrial cancer. A number of serum biomarkers, including human epididymis protein 4 (HE4) [34] and cancer antigen 125 (CA-125) [35] have been studied in endometrial cancer, but their low diagnostic performance limits their clinical utility. Multibiomarker panels may have superior discriminatory power compared with individual biomarkers [32,36]. For instance, transferrin, prealbumin and apolipoprotein-1 combined enable the early detection of endometrial cancer with a sensitivity and specificity of 71% and 88%, and detection of late stage disease with a sensitivity of 82% and a specificity of 86%, respectively [36]. Circulating tumor DNA (ctDNA) is a useful prognostic biomarker for endometrial cancer but its low levels in pre-symptomatic and early stage disease limit its use as a screening tool [37]. Other potential non-invasive diagnostic blood biomarkers for endometrial cancer include microRNAs. Small pilot studies have shown specific microRNAs are variously up- or down-regulated in cancer cases compared to controls [38,39,40]. These molecular tests may facilitate endometrial cancer detection. However, their mediocre accuracy and/or high costs limit their use in the clinic. Spectroscopy is based on an inexpensive multiuse platform, gives an immediate result and is a potential alternative to other costly and laborious assays under development. A previous pilot study by our group (including 30 endometrial cancers and 30 healthy controls) revealed the potential of vibrational spectroscopy to detect endometrial cancer using blood serum, achieving an accuracy of 82% overall [15].

To our knowledge, this is the largest study to date that explores the potential of blood spectroscopy as an early detection tool in endometrial cancer. Patient characteristics were prospectively recorded and included in our analysis to identify and control for potential confounding factors, where previous studies did not. All healthy control participants were investigated by ultrasound scan and/or endometrial biopsy to exclude pathology at baseline and were followed up for at least 12 months to ensure their reliability as ‘controls’, another strength of our work. A potential limitation of spectroscopy is that the derived peaks can only be tentatively assigned to biomolecules because spectral regions are formed by many different biological entities; this limits its utility at unpicking the exact molecular pathways involved in carcinogenesis. On the other hand, spectroscopic techniques provide a disease signature and can reveal information about the status of a sample being either pathological or healthy in a snapshot. Replication of our results in even larger cohorts and in high risk groups, such as women with significant obesity [41], and those with inherited predisposition syndromes, for example Lynch syndrome [42], is an important consideration for the future validation of spectroscopy for endometrial cancer detection. Future studies of asymptomatic women at high risk of endometrial cancer followed up longitudinally are needed to establish whether spectroscopic markers can predict the emergence of cancer after negative gold-standard endometrial assessment.

A spectroscopic blood test could serve multiple roles in the current clinical workflow, either independently or within algorithms that combine other biomarker tests. Scenarios include the initial investigation of women with unexplained postmenopausal bleeding; screening asymptomatic women with incidental radiological evidence of endometrial thickening; screening high-risk women, particularly obese women, in whom ultrasound might be problematic; alerting pathologists and clinicians to the histological type of endometrial cancer; and monitoring disease managed by uterine-sparing protocols [43]. Additionally, this simple blood test might be useful in developing countries, where costly molecular and imaging technologies are not readily available. Portable and hand-held infrared spectrometers are already being trialled for point-of-care testing in developing countries to detect diseases in blood, for example, malaria [44].

In this study, we have clearly demonstrated that blood biospectroscopy can differentiate early stage 1 cancers from controls and therefore detect disease when there is a high chance of therapeutic cure. Further studies are required to fully evaluate the technique’s potential at discriminating pre-invasive disease from cancers particularly as the subjective nature of classifying pre-invasive disease can lead to misclassification of original disease phenotype.

In England, the NHS devised a strategy in 2016 to fast track all people with suspected cancer by improving patient pathways, facilitating early access to medical care and the establishment of rapid diagnostic and assessment centres. Incorporation of a simple, low-cost blood test that gives an instantaneous result could potentially triage patients so that secondary care is not overwhelmed by healthy women and only those women at significant risk of disease are referred for invasive diagnostic workup. The new NHS standards to be enforced by 2020 set out that diagnosis should be established within 28 days of attending primary care; this can only be achieved through the introduction of innovative solutions. Spectroscopy is an emerging technology with potential to deliver on this ambitious target.

## 4. Materials and Methods

### 4.1. Study Design

The primary objective of the study was to assess the ability of infrared spectroscopy to detect women with endometrial cancer at its earliest stage as well as its precursor lesion, atypical hyperplasia, using blood samples. The secondary objective was to determine whether spectroscopy could discriminate between Type I and Type II cancers. Women were recruited from clinics at Manchester University NHS Foundation Trust, Salford Royal Foundation Trust and Lancashire Teaching Hospitals if they were undergoing investigation for unexplained postmenopausal bleeding, investigation and treatment for atypical hyperplasia or endometrial cancer, surgery for benign gynaecological conditions or management of obesity. All women gave written, informed consent to participate and donated their clinical data and blood samples for future research. Ethical approval was obtained as follows: Weight loss study (North West Research Ethics Committee ref. 12/NW/0050), PROTEC study (Cambridge East Research Ethics Committee ref. 15/EE/0063), PREMIUM study (North West Research Ethics Committee ref. 14/NW/1236), Metformin study (North West Research Ethics Committee ref. 11/NW/0442), PETALS study (NRES Committee North West—Lancaster ref. 15/NW/0733), DETECT study (North West Research Ethics Committee—Greater Manchester 16/NW/0660) and East of England—Cambridge Central Research Ethics Committee ref. 16/EE/0010. Cases and controls were evenly distributed across the different clinics, studies and recruitment period. Blood was taken in clinic or on the day of surgery; the latter group of patients had fasted for at least six hours prior to blood draw. Women undergoing bariatric surgery (gastric sleeve or bypass) had additionally followed a special low-calorie diet prior to surgery (the so-called ‘liver diet’) to reduce the risk of complications at laparoscopy. Blood samples were collected, processed and stored as described below. Most women underwent hysterectomy for endometrial cancer, atypical hyperplasia or benign gynaecological indications. All other women had endometrial biopsies taken in clinic or under general anaesthesia (e.g., at the time of bariatric surgery) using a Pipelle endometrial sampler, to confirm the absence of pathology. The exception to this was women referred with postmenopausal bleeding whose endometrial thickness was <4 mm on transvaginal ultrasound scan. Endometrial biopsies were assessed by specialist gynaecological pathologists. Only women with normal endometrium at the time of blood draw (by biopsy and/or scan) and for ≥12 months afterwards, were included as healthy controls. All hysterectomy specimens showing atypical endometrial hyperplasia or cancer were assessed by at least two specialist gynaecological pathologists reporting to Royal College of Pathology Standards. Hysterectomy specimens from women with presumed atypical hyperplasia were examined in their entirety to exclude co-existing cancer. Some women with atypical hyperplasia or cancer were managed conservatively; these cases were all discussed at the Specialist Gynaecological Oncology Multidisciplinary Team Meeting following review by at least two consultant gynaecological pathologists with a specialist interest in endometrial pathology. Only cases with two consecutive pre-treatment biopsies (oral or intra-uterine progestin) showing atypical hyperplasia only and no co-existing cancer were included as cases of atypical hyperplasia. Type I endometrial cancers were pure endometrioid adenocarcinomas whereas Type II included serous, clear cell and carcinosarcomas; the mixed group included cases with different subtypes. Atypical hyperplasia was diagnosed according to WHO reporting standards [45].

Demographic details, including age, body mass index (BMI), diabetes and blood pressure, were recorded and taken into account as potential confounding factors. Participants were categorized into two groups according to age, using the mean age at blood draw as our cut-off (60 years). Women were categorized according to their BMI into the following groups: underweight (BMI < 18 kg/m^2^), normal weight (BMI = 18.5–24.9 kg/m^2^), overweight (BMI = 25–29.9 kg/m^2^), obese (BMI = 30–39.9 kg/m^2^) and severely obese (BMI > 40 kg/m^2^). Fasting status and use of the pre-bariatric surgery liver diet were recorded and included as potential confounding factors. Missing data were recorded for a small proportion of women: BMI 3/652 (<1%), diabetic status 3/652 (<1%), blood pressure 83/652 (13%) and fasting status 97/652 (15%), respectively. In total, 83/652 (13%) followed the liver diet for three weeks prior to blood draw. The researcher who performed the spectroscopic analysis (MP) was blinded to clinical information and histological results during collection of spectroscopic data. The results of the spectroscopic test were not available to the pathologists at the time of histological assessment.

### 4.2. Sample Preparation and Spectroscopic Analysis

Blood was collected in standard EDTA tubes, centrifuged at 2000 rpm for 10 min to remove the cells and the supernatant (i.e., plasma) was collected into microtubes and stored at −80 °C until analysis. Frozen samples were thawed, 50 μL was deposited onto IR-reflective glass slides (MirrIR Low-E slides, Kevley Technologies) and left to air-dry at room temperature.

Spectra were collected with attenuated total reflection Fourier-transform infrared (ATR-FTIR) spectroscopy using a Tensor 27 FTIR spectrometer with a Helios ATR attachment containing a diamond ATR crystal (Bruker Optics Ltd., Coventry, UK). In this setting, the ATR crystal is on the top of the attachment and the slide with the sample is placed on the platform with the sample facing up; the platform is then moved upward to ensure good contact with the crystal [46]. Spectral resolution was 8 cm^−1^ with 2× zero-filling, giving a data-spacing of 4 cm^−1^. Thirty-two co-additions and a mirror velocity of 2.2 kHz were used for optimum signal-to-noise ratio. A closed-circuit television (CCTV) camera attachment was used to locate the area of interest and spectra were acquired from five different locations of the blood spot to minimize bias. The diamond crystal was cleaned with distilled water and dried before the next sample; a background spectrum was taken after the analysis of each sample to account for potential changes in ambient conditions.

### 4.3. Data Analysis

Pre-processing of the acquired spectra is an essential step of all spectroscopic experiments and is used to correct problems associated with spectral acquisition and instrumental noise. All data processing was performed within MATLAB R2014b environment (MathWorks Inc., Natick, MA, USA). An in-house developed IRootLab toolbox (http://trevisanj.github.io/irootlab/) was implemented to load and pre-process the data, prior to which spectra were averaged by five to account for differences between participants rather than individual spectra. Model construction was performed using the PLS Toolbox version 7.9.3 (Eigenvector Research Inc., Manson, WA, USA). Spectra were cut at the fingerprint region (1800–900 cm^−1^), followed by 2nd Savitzky–Golay (SG) derivative (window of 5 points, 2nd-order polynomial fitting) and vector normalization.

Samples were divided into training (70%) and test (30%) datasets before further multivariate analysis by using the Kennard–Stone uniform sample selection algorithm (Figure S3). Partial least squares discriminant analysis (PLS-DA) is a widely used supervised classification approach based on a linear classification model. PLS is applied to the pre-processed data to reduce the original variables to a few number of latent variables (LVS) containing scores and loadings. Both spectral information and patient groups are used in PLS decomposition, where the predicted response is obtained through a vector of regression coefficients; a linear classifier is then applied to divide the predicted response into groups. For the purposes of this study, PLS-DA was performed on a sample/patient basis with cross-validation (venetian blinds with 10 data splits) used to select the number of LVS (Figure S4). Six discriminatory peaks were identified for each pairwise comparison based on the PLS-DA regression coefficients according to the absolute highest or lowest weights (Figure S3). Discriminatory peaks were detected and evaluated after 2nd differentiation; the differences in the absorbance levels of these peaks were calculated after automatic weighted least squares baseline correction and vector normalization, as the 2nd differentiation changes the spectral scale to coefficient values rather than absorbance. ROC curves and area under the curve (AUC) values were calculated using the easyROC version 1.3.1 (http://www.biosoft.hacettepe.edu.tr/easyROC/). AUC values represent the accuracy of the test with values ranging from 0.9 to 1 being considered excellent, 0.8 to 0.9 good and 0.7 to 0.8 fair.

Principal component analysis (PCA) is an unsupervised method of exploratory analysis that reduces the spectral dataset to only a small number of factors, principal components (PCs). Each PC is orthogonal to each other and covers most of the variance present in the original data so that the first PC covers the largest explained variance, followed by the second PC and so on. Each PC is composed of scores (projections of the samples on the PC direction) and loadings (angle cosines of the wavenumbers projected on the PC direction). The score plots can be generated to visualize the differences and similarities between the groups. PCA was performed to investigate the effect of potential confounding factors, such as age (<60 years; ≥60 years), BMI (normal: BMI = 18.5–24.9; overweight: BMI = 25–29.9; obese: BMI = 30–39.9; severely obese: BMI > 40), diabetes (diabetic; non-diabetic), fasting status (fasting; non-fasting; liver diet) and blood pressure (normotension; hypertension). PCA is preferred to investigate variations within groups as, in contrast to the supervised PLS-DA, it requires no prior knowledge of the disease class and thus it shows the “natural variations” within a dataset.

### 4.4. Statistical Analysis

Statistical analysis was performed within MATLAB. All statistical tests were performed on a patient basis using the pre-processed data (2nd SG derivative and vector normalization) within the biofingerprint region. Significant *p*-values were obtained through ANOVA tests applied to each individual spectral marker response or MANOVA tests applied to the whole pre-processed spectra for the confounding factor comparisons. A *p*-value <0.05 was considered significant. *p*-values were also calculated to test for differences between the clinical characteristics of the groups (controls versus reference class) using a *t*-test for the age comparison (data input as mean and standard deviation) and a Pearson’s chi-squared test of independence for the other parameters (BMI, diabetes, blood pressure and fasting status) based on a cross-tabulation analysis with the population size of each group. Unknowns were excluded from the analysis to avoid inclusion of undefined sources of variation in the test. For sample size calculations, a power test using a two-tailed *t*-test (data input as mean and standard deviation in absorbance units of the spectral data for each class) indicated a minimum number of 94 control samples, 151 cancer samples and 49 hyperplasia samples for a power of 80%. In this study, we included more than the required number of samples (242 controls, 342 endometrial cancers and 68 atypical endometrial hyperplasia) to ensure our conclusions were robust.

## 5. Conclusions

We demonstrate the use of a blood-based test as a non-invasive and inexpensive approach to effectively diagnose endometrial cancer and therefore triage women for invasive biopsy. This is the largest study of its kind to demonstrate that spectroscopy of blood plasma can serve as an early detection tool in endometrial cancer, hugely improving prognostic outcomes and expediting therapeutic intervention. Women affected by or at risk of endometrial cancer will be the ultimate beneficiaries of such a test, for whom few advances have been made in recent years despite the rising incidence and deaths from the disease. This offers an important step forward for patients, clinicians and the research community and has the potential to become practice changing.

## Figures and Tables

**Table 1:** Patient characteristics.

Patient Characteristics	Controls (*n* = 242)	Cancer (*n* = 342)			Atypical Hyperplasia (*n* = 68)	All (*n* = 652)
		Type I (*n* = 258)	Type II (*n* = 64)	Mixed * (*n* = 20)		
**Age, years**						***p*** **-value: 0.268**
Mean (SD)	52 (11)	63 (13)	69 (10)	69 (10)	52 (15)	-
<60, n/N (%)	194/242 (80)	92/258 (36)	10/64 (16)	4/20 (20)	45/68 (66)	345/652 (53)
≥60, n/N (%)	48/242 (20)	166/258 (64)	54/64 (84)	16/20 (80)	23/68 (34)	307/652 (47)
*p*-value	-	<0.0001	<0.0001	<0.0001	1.00	-
**BMI, *n*/*N* (%)**						***p*** **-value: 0.412**
Underweight (<18)	0/242 (0)	1/258 (0)	2/64 (3)	0/20 (0)	0/68 (0)	3/652 (<1)
Normal weight (18.5–24.9)	33/242 (14)	33/258 (13)	17/64 (26.5)	2/20 (10)	1/68 (1)	86/652 (13)
Overweight (25–29.9)	42/242 (17)	58/258 (22)	17/64 (26.5	6/20 (30)	5/68 (7)	128/652 (20)
Obese (30–39.9)	41/242 (17)	95/258 (37)	20/64 (31)	8/20 (40)	14/68 (21)	178/652 (27)
Severely obese (>40)	124/242 (51)	71/258 (28)	7/64 (11)	4/20 (20)	48/68 (71)	254/652 (39)
Unknown	2/242 (1)	0/258 (0)	1/64 (2)	0/20 (0)	0/68 (0)	3/652 (<1)
*p*-value	-	0.220	0.241	0.220	0.220	-
**Diabetes, *n*/*N* (%)**						***p*** **-value: 0.268**
Yes	58/242 (24)	47/258 (18)	9/64 (14)	4/20 (20)	21/68 (31)	139/652 (21)
No	184/242 (76)	210/258 (81)	54/64 (84)	15/20 (75)	47/68 (69)	510/652 (78)
Unknown	0/242 (0)	1/258 (<1)	1/64 (2)	1/20 (5)	0/68 (0)	3/652 (<1)
*p*-value	-	0.157	0.157	0.157	0.157	-
**Blood pressure, *n*/*N* (%)**						***p*** **-value: 0.268**
Normotension	128/242 (53)	129/258 (50)	38/64 (59)	9/20 (45)	30/68 (44)	334/652 (51)
Hypertension	74/242 (31)	108/258 (42)	14/64 (22)	3/20 (15)	36/68 (53)	235/652 (36)
Unknown	40/242 (16)	21/258 (8)	12/64 (19)	8/20 (40)	2/68 (3)	83/652 (13)
P-value	-	0.157	0.157	0.157	0.157	-
**Fasting status, *n*/*N* (%)**						***p*** **-value: 0.345**
Fasting	36/242 (15)	188/258 (73)	36/64 (56)	7/20 (35)	43/68 (63)	310/652 (47)
Non-fasting	93/242 (38)	38/258 (15)	12/64 (19)	3/20 (15)	16/68 (24)	162/652 (25)
Liver diet	73/242 (30)	3/258 (1)	0/64 (0)	0/20 (0)	7/68 (10)	83/652 (13)
Unknown	40/242 (17)	29/258 (11)	16/64 (25)	10/20 (50)	2/68 (3)	97/652 (15)
*p*-value	-	0.199	0.199	0.199	0.199	-

* Mixed includes endometrioid and clear cell, high-grade serous and clear cell, endometrioid with serous component, endometrioid with squamous component, endometrioid carcinosarcoma. SD: standard deviation; BMI: body mass index.

## Supplementary Materials

The following are available online at https://www.mdpi.com/2072-6694/12/5/1256/s1, Figure S1: Raw infrared spectra in the biofingerprint region (1800–900 cm^−1^) for control, Type I, Type II and hyperplasia samples. Figure S2: Assessment of the quality of the samples collected from the biobanks at Central Manchester or Lancashire Teaching Hospitals. Figure S3: Partial least squares discriminant analysis (PLS-DA) regression coefficients and correct classification rates. Samples were split into training (70%) and test (30%) datasets. Figure S4: Cross-validation (CV) error rate varying according to the number of latent variables for partial least squares discriminant analysis (PLS-DA). Figure S5: Partial least squares discriminant analysis (PLS-DA) results for hyperplasia vs. Type I, Type II, and hyperplasia. Figure S6: Receiver operating characteristic (ROC) curves along with correct classification rates for the training, cross validation and test datasets. Figure S7: Mean pre-processed spectra for all the subgroup comparisons. Table S1: Mean and standard deviation (SD) as absorbance units for the selected features by partial least squares discriminant analysis (PLS-DA) for the different comparisons (A) to (G).
