# Supplementary material for: Detecting Endometrial Cancer by Blood Spectroscopy: A Diagnostic Cross-Sectional Study
Source: Cancers (Basel). 2020 May 16;12(5):1256. doi: 10.3390/cancers12051256 (PMC7281323; doi:10.3390/cancers12051256)
Supplement: Supplementary file 1 [file cancers-12-01256-s001.pdf]

Supplementary Material: Detecting endometrial cancer by blood spectroscopy: A diagnostic cross-sectional study

Maria Paraskevaidi, Camilo L.M. Morais, Katherine M. Ashton, Helen F. Stringfellow, Rhona J. McVey, Neil A.J. Ryan, Helena O'Flynn, Vanitha N. Sivalingam, Sarah J. Kitson, Michelle L. MacKintosh, Abigail E. Derbyshire, Cecilia Pow, Olivia Raglan, Kássio M.G. Lima, Maria Kyrgiou, Pierre L. Martin-Hirsch, Francis L. Martin, Emma J. Crosbie

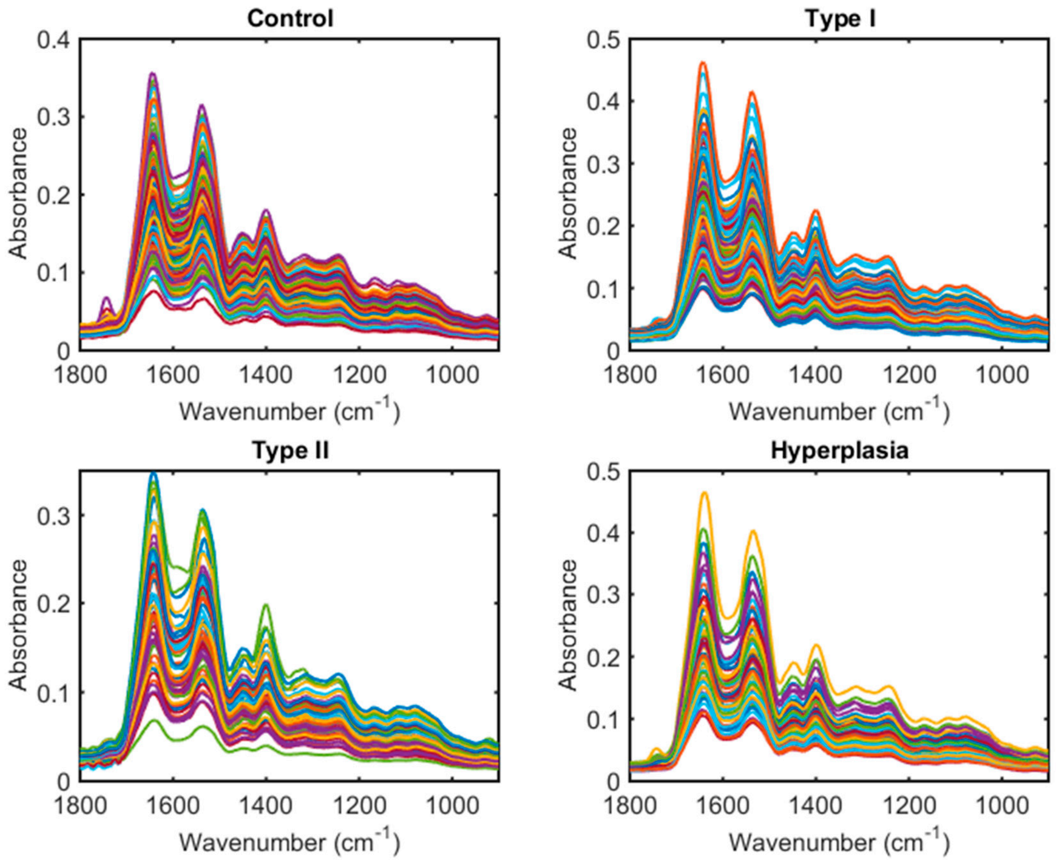

Figure S1. Raw infrared spectra in the biofingerprint region (1800-900 cm^{-1}) for control, Type I, Type II and hyperplasia samples.

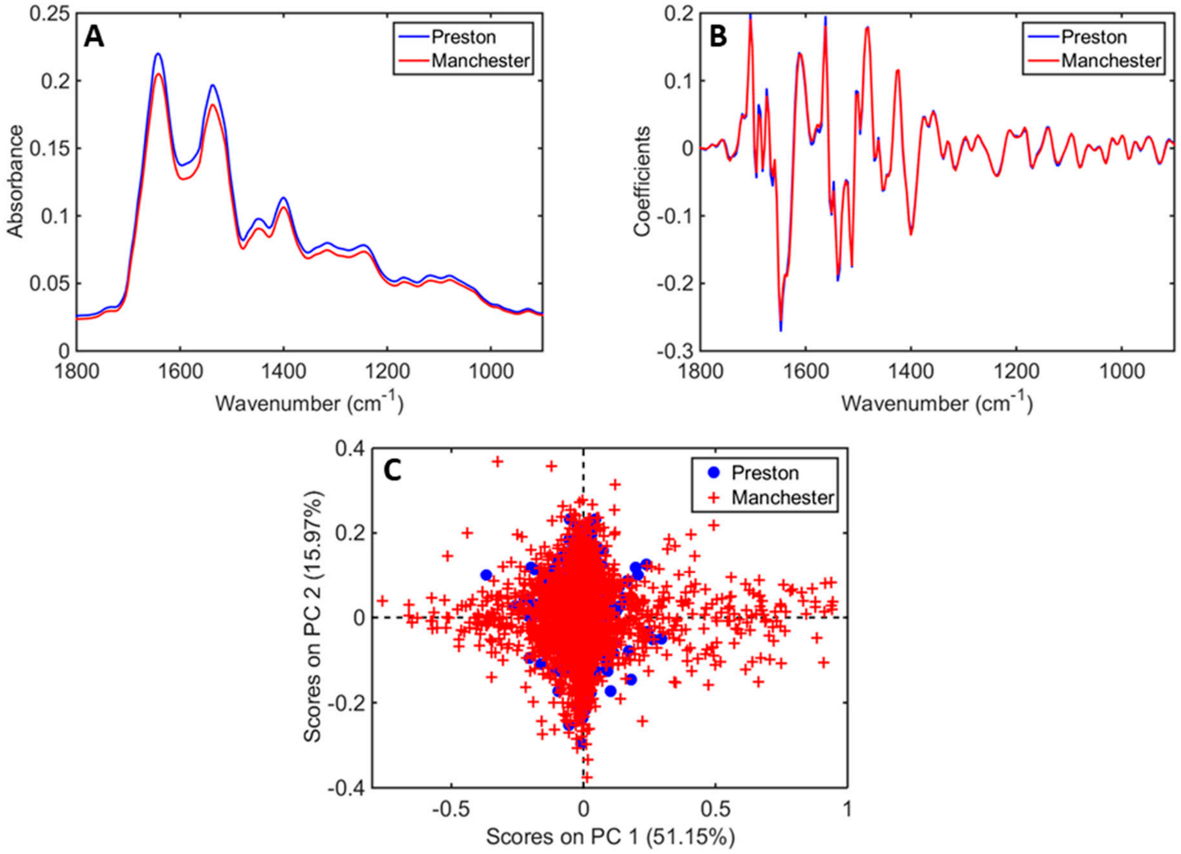

Figure S2. Assessment of the quality of the samples collected from the biobanks at Central Manchester or Lancashire Teaching Hospitals. (**A**) Average raw infrared spectra. (**B**) Pre-processed spectra (Savitzky-Golay 2nd derivative and vector normalisation) (**C**) Scores plot after principal component analysis (PCA) of the pre-processed spectra (p = 0.973).

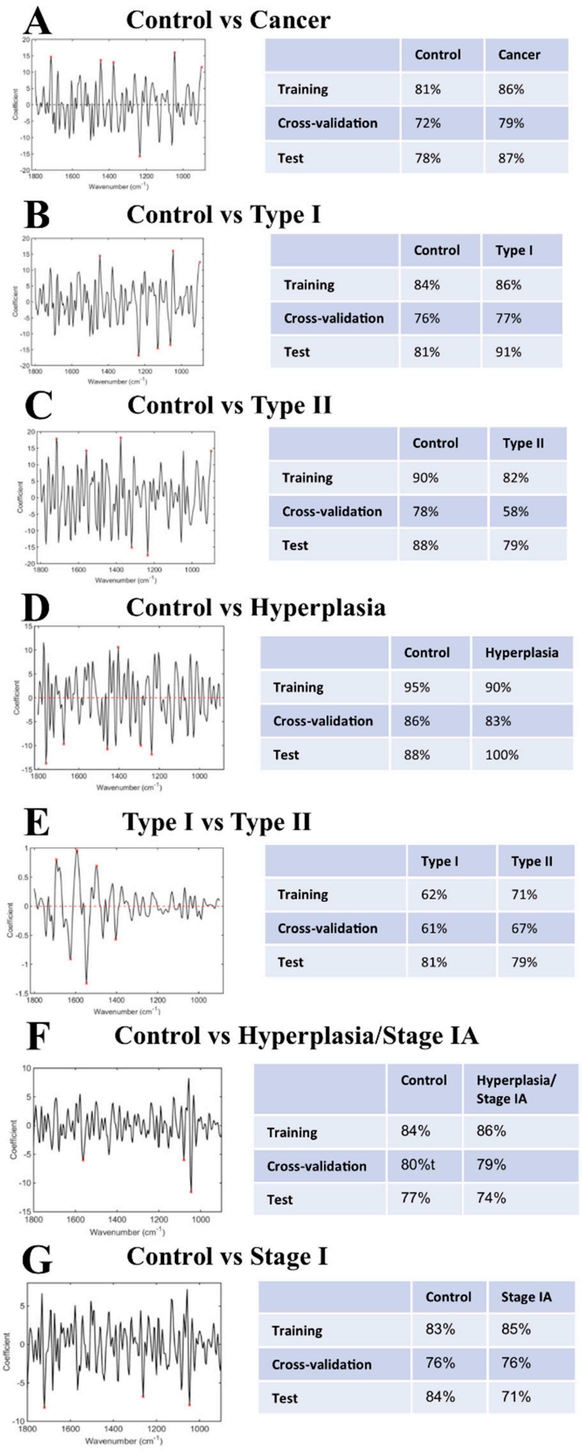

A Control vs Cancer

	Control	Cancer
Training	81%	86%
Cross-validation	72%	79%
Test	78%	87%

B Control vs Type I

	Control	Type I
Training	84%	86%
Cross-validation	76%	77%
Test	81%	91%

C Control vs Type II

	Control	Type II
Training	90%	82%
Cross-validation	78%	58%
Test	88%	79%

D Control vs Hyperplasia

	Control	Hyperplasia
Training	95%	90%
Cross-validation	86%	83%
Test	88%	100%

E Type I vs Type II

	Type I	Type II
Training	62%	71%
Cross-validation	61%	67%
Test	81%	79%

F Control vs Hyperplasia/Stage IA

	Control	Hyperplasia/ Stage IA
Training	84%	86%
Cross-validation	80%t	79%
Test	77%	74%

G Control vs Stage I

	Control	Stage IA
Training	83%	85%
Cross-validation	76%	76%
Test	84%	71%

Figure S3. Partial least squares discriminant analysis (PLS-DA) regression coefficients and correct classification rates. Samples were split in training (70%) and test (30%) datasets. (**A**) Control (*n* = 242) vs cancer (*n* = 342; including Type I (*n* = 258), Type II (*n* = 64) and mixed (*n* = 20)). (**B**) Control (*n* = 242) vs Type I cancers (*n* = 258). (**C**) Control (*n* = 242) vs Type II (*n* = 64) cancers. (**D**) Control (n = 242) vs Hyperplasia (*n* = 68). (**E**) Type I (*n* = 258) vs Type II cancers (*n* = 64). (**F**) Control (*n* = 242) vs Hyperplasia/Stage IA (*n* = 260). (G) Control (*n* = 242) vs Stage I (*n* = 254).

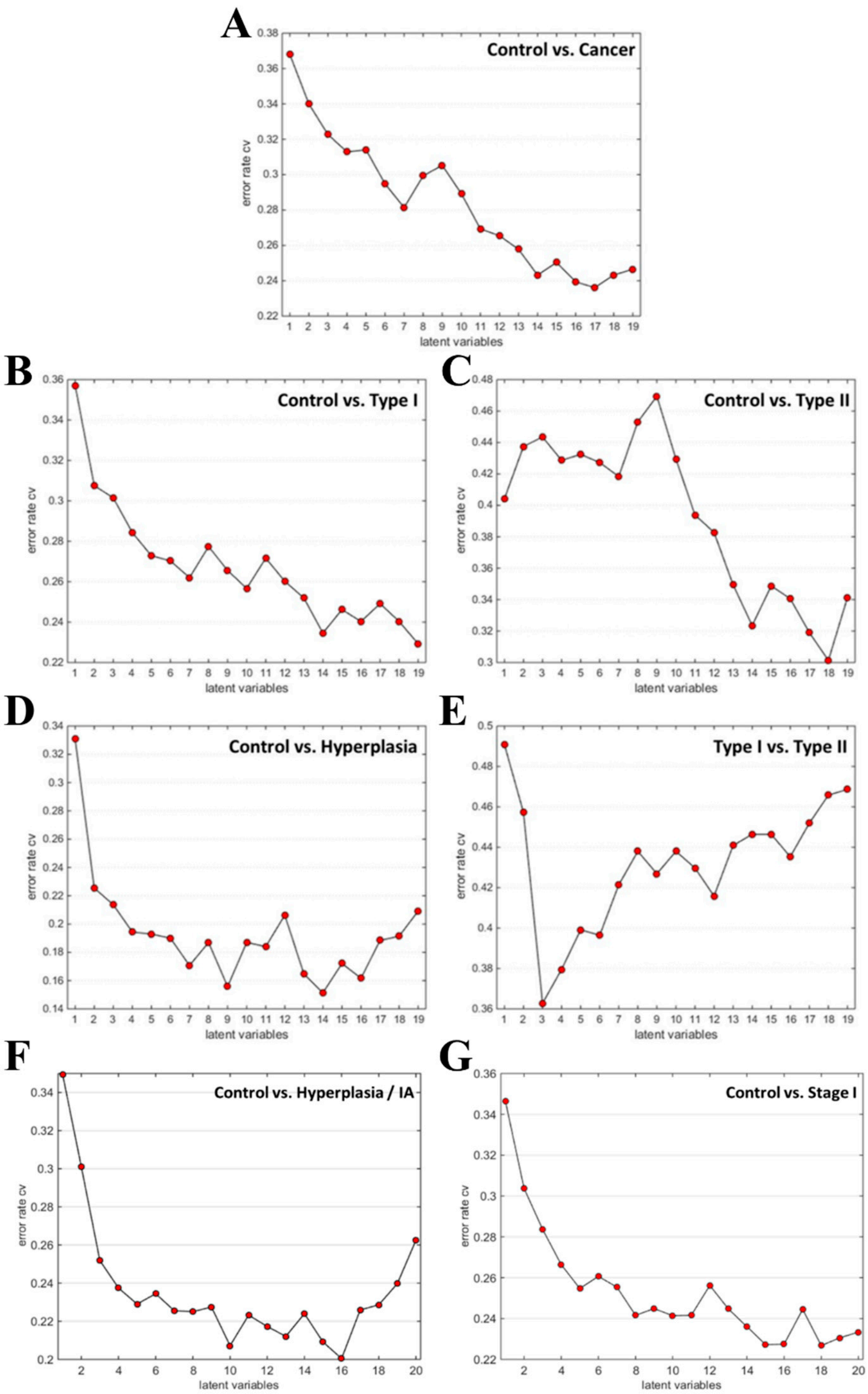

Figure S4. Cross-validation (CV) error rate varying according to the number of latent variables for partial least squares disciminant analysis (PLS-DA). (**A**) Control (*n* = 242) vs cancer (*n* = 342; including Type I (*n* = 258), Type II (*n* = 64) and mixed (*n* = 20)), 14 selected LVs (97% explained variance). (**B**) Control (*n* = 242) vs Type I cancers (*n* = 258), 14 selected LVs (97% explained variance). (**C**) Control (n = 242) vs Type II (*n* = 64) cancers, 14 selected LVs (97% explained variance). (**D**) Control (*n* = 242) vs Hyperplasia (*n* = 68), 14 selected LVs (97% explained variance). (**E**) Type I (*n* = 258) vs Type II cancers (*n* = 64), 3 selected LVs (72% explained variance). (**F**) Control (*n* = 242) vs Hyperplasia/Stage IA (n = 260), 10 selected LVs (93% explained variance). (**G**) Control (*n* = 242) vs Stage I (*n* = 254), 10 selected LVs (94% explained variance).

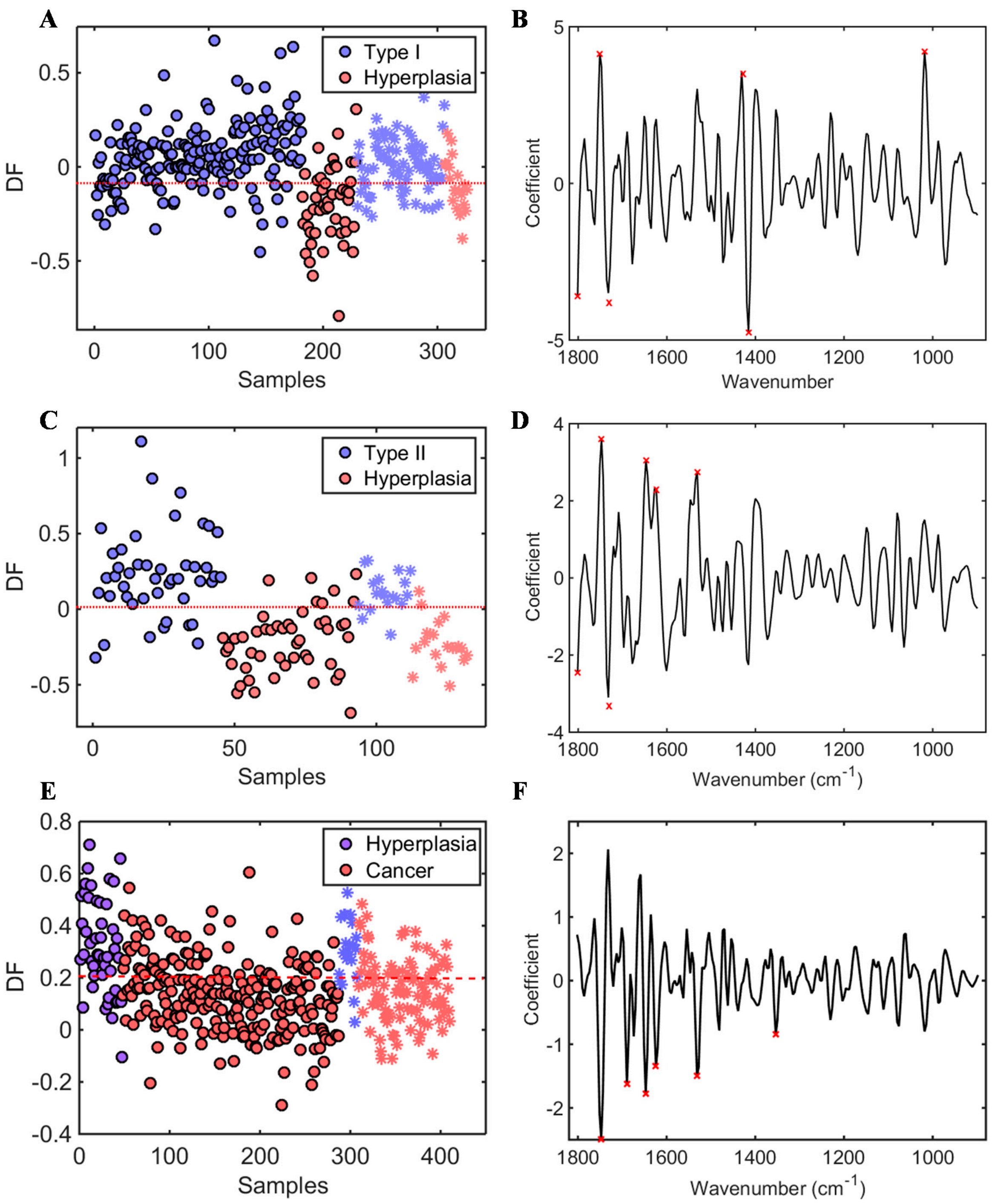

Figure S5. Partial least squares disciminant analysis (PLS-DA) results for hyperplasia vs. Type I, Type II, and Hyperplasia. (**A**) Discriminant function (DF) plot for Type I vs Hyperplasia. (**B**) PLS-DA regression coefficients for Type I vs Hyperplasia. (**C**) Discriminant function (DF) plot for Type II vs Hyperplasia. (**D**) PLS-DA regression coefficients for Type II vs Hyperplasia. (**E**) Discriminant function (DF) plot for Hyperplasia vs Cancer. (F) PLS-DA regression coefficients for Hyperplasia vs Cancer. Inset: o = training samples, * = test samples.

A **Hyperplasia vs Type I**

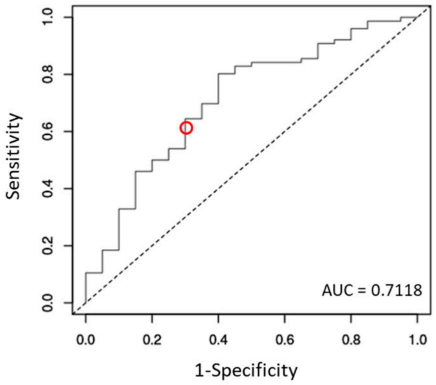

	Correct classification	
	Type I	Hyperplasia
Training	82%	71%
Cross-validation	77%	63%
Test	77%	60%
**Accuracy**	**Sensitivity**	**Specificity**
73%	60%	77%

B **Hyperplasia vs Type II**

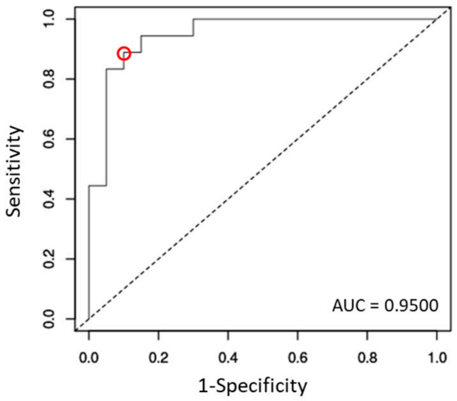

	Correct classification	
	Type II	Hyperplasia
Training	82%	85%
Cross-validation	71%	81%
Test	84%	90%
**Accuracy**	**Sensitivity**	**Specificity**
87%	90%	84%

C **Hyperplasia vs Cancer**

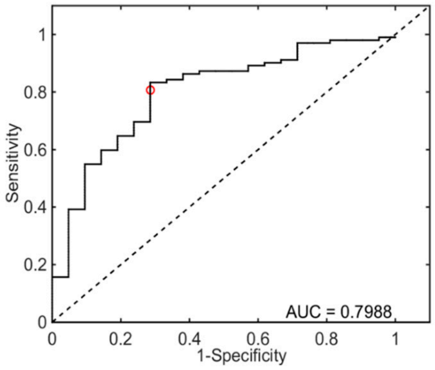

	Correct classification	
	Hyperplasia	Cancer
Training	66%	82%
Cross-validation	62%	79%
Test	71%	80%
**Accuracy**	**Sensitivity**	**Specificity**
76%	80%	71%

Figure S6. Receiver operating characteristic (ROC) curves along with correct classification rates for the training, cross validation and test datasets. Overall accuracies, sensitivities, specificities and area under the curve (AUC) values are also shown. (**A**) Hyperplasia vs Type I (**B**) Hyperplasia vs Type II (**C**) Hyperplasia vs cancer.

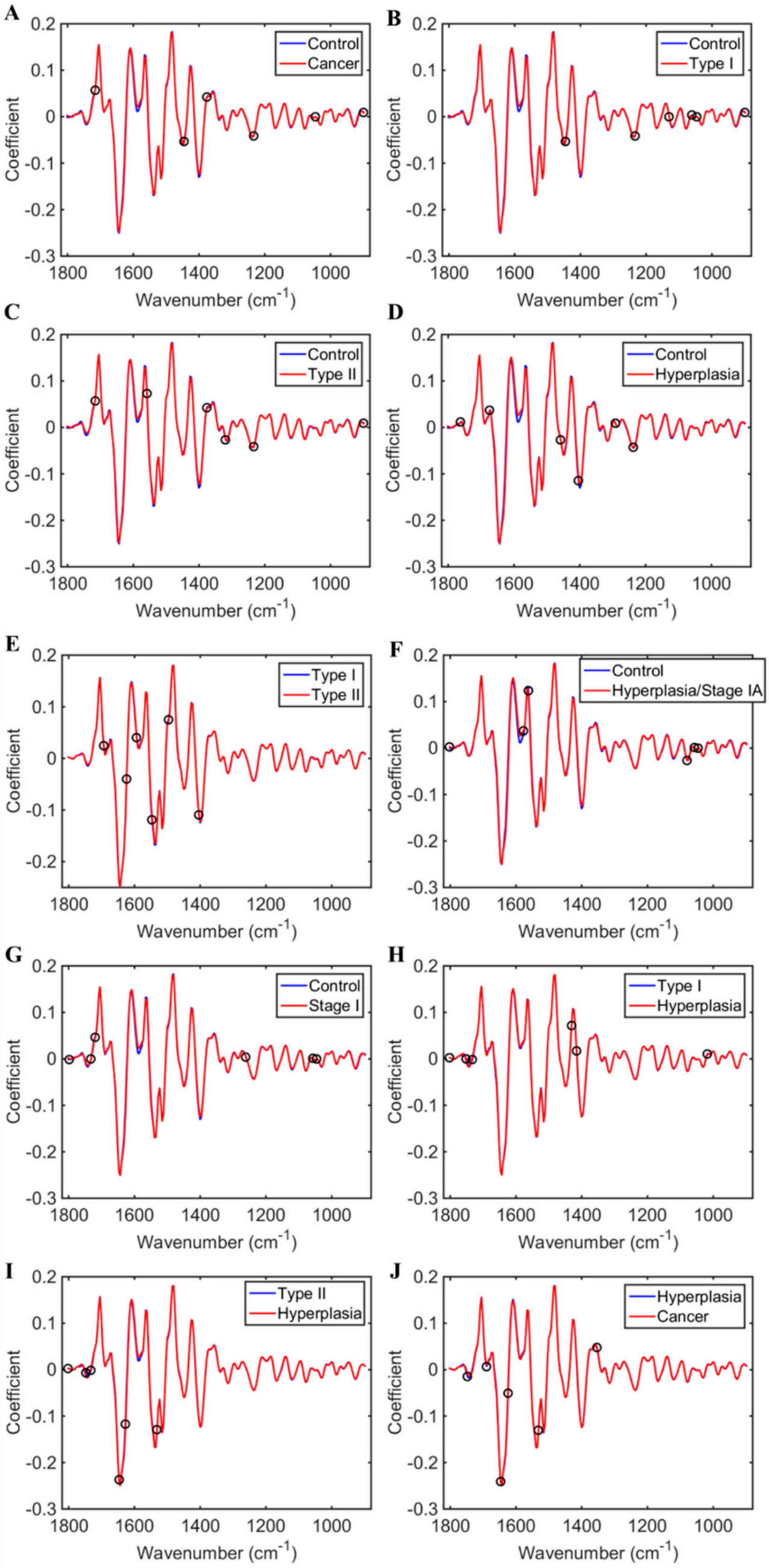

Figure S7. Mean pre-processed spectra for all the subgroup comparisons **(A)** Control vs cancer. **(B)** Control vs Type I. **(C)** Control vs Type II. **(D)** Control vs Hyperplasia. **(E)** Type I vs Type II. **(F)** Control vs Hyperplasia/Stage IA. **(G)** Control vs Stage I. **(H)** Type I vs Hyperplasia. **(I)** Type II vs Hyperplasia. **(J)** Hyperplasia vs cancer. Circled regions represent wavenumbers with the highest absolute regression coefficients by PLS-DA (see Table S1).

Table S1. Mean and standard-deviation (SD) as absorbance units for the selected features by partial least squares disciminant analysis (PLS-DA) for the different comparisons (A) to (G).

**(A)**	**Control (1) vs Cancer (2)**	**Class 1**	**Class 2**	**SD 1**	**SD 2**
	1716 cm^{-1}	−0.003	−0.0029	0.0012	0.0015
	1446 cm^{-1}	0.0388	0.0393	0.0016	0.0011
	1377 cm^{-1}	0.0276	0.0264	0.0035	0.0016
	1234 cm^{-1}	0.0154	0.0155	0.0012	0.001
	1045 cm^{-1}	5.00E −03	5.20E −03	0.0018	0.0015
	900 cm^{-1}	0.0091	0.0088	0.0012	0.0008
**(B)**	**Control (1) vs Type I (2)**				
	1446 cm^{-1}	0.0388	0.0392	0.0016	0.001
	1234 cm^{-1}	0.0154	0.0155	0.0012	0.001
	1130 cm^{-1}	−2.30E −03	−2.30E −03	0.0006	0.0005
	1061 cm^{-1}	0.0062	0.0065	0.0016	0.0013
	1045 cm^{-1}	5.00E −03	5.10E −03	0.0018	0.0014
	900 cm^{-1}	0.0091	0.0088	0.0012	0.0008
**(C)**	**Control (1) vs Type II (2)**				
	1715 cm^{-1}	−0.003	−0.0028	0.0012	0.002
	1559 cm^{-1}	0.121	0.121	0.0027	0.0041
	1377 cm^{-1}	0.0276	0.0268	0.0035	0.0023
	1319 cm^{-1}	0.0168	0.0161	0.0024	0.0019
	1234 cm^{-1}	0.0154	0.0156	0.0012	0.0011
	900 cm^{-1}	0.0091	0.0089	0.0012	0.0008
**(D)**	**Control (1) vs Hyperplasia (2)**				
	1763 cm^{-1}	−0.002	−0.0019	0.0006	0.0004
	1674 cm^{-1}	0.1049	0.1047	0.0037	0.0035
	1458 cm^{-1}	0.0353	0.0358	0.0024	0.0011
	1404 cm^{-1}	0.0575	0.0547	0.006	0.0032
	1292 cm^{-1}	0.0121	0.0118	0.0012	0.0009
	1238 cm^{-1}	0.017	0.0171	0.0011	0.0009
**(E)**	**Type I (1) vs Type II (2)**				
	1693 cm^{-1}	0.0421	0.0434	0.0026	0.0028
	1624 cm^{-1}	0.163	0.1623	0.0036	0.0037
	1593 cm^{-1}	0.0975	0.0985	0.0055	0.0067
	1547 cm^{-1}	0.1518	0.15	0.0027	0.0023
	1497 cm^{-1}	0.06	0.0611	0.0033	0.0037
	1404 cm^{-1}	0.0554	0.0553	0.0036	0.0049
**(F)**	**Control (1) vs Hyperplasia/Stage IA (2)**				
	1800 cm^{-1}	0.0049	0.005	0.0012	0.0011
	1578 cm^{-1}	0.0991	0.1016	0.0045	0.0069
	1562 cm^{-1}	0.1114	0.111	0.0027	0.003
	1080 cm^{-1}	0.0078	0.0076	0.0013	0.0016
	1057 cm^{-1}	0.0061	0.006	0.0014	0.0016
	1045 cm^{-1}	0.0051	0.005	0.0015	0.0018
**(G)**	**Control (1) vs Stage I (2)**				
	1800 cm^{-1}	0.0048	0.005	0.0013	0.0011
	1732 cm^{-1}	−0.0025	−0.0027	9.15E −04	9.79E −04
	1720 cm^{-1}	−0.0041	−0.0044	6.10E −04	6.23E −04
	1261 cm^{-1}	0.0139	0.014	8.29E −04	8.66E −04
	1057 cm^{-1}	0.0062	0.006	0.0013	0.0016
	1045 cm^{-1}	0.0051	0.005	0.0014	0.0018
**(H)**	**Type I (1) vs Hyperplasia (2)**				
	1800 cm^{-1}	0.0241	0.0246	0.0037	0.0036
	1751 cm^{-1}	0.0280	0.0280	0.0053	0.0054
	1732 cm^{-1}	0.0305	0.0304	0.0059	0.0059
	1431 cm^{-1}	0.0880	0.0851	0.0247	0.0235
	1416 cm^{-1}	0.0971	0.0936	0.0280	0.0268
	1018 cm^{-1}	0.0396	0.0384	0.0101	0.0092
**(I)**	**Type II (1) vs Hyperplasia (2)**				
	1800 cm^{-1}	0.0246	0.0227	0.0036	0.0033
	1747 cm^{-1}	0.0290	0.0280	0.0061	0.0056

	1732 cm $^{-1}$	0.0304	0.0292	0.0059	0.0054
	1647 cm $^{-1}$	0.2016	0.2198	0.0629	0.0747
	1628 cm $^{-1}$	0.1844	0.2032	0.0570	0.0697
	1531 cm $^{-1}$	0.1783	0.1946	0.0550	0.0657
**(J)**	**Hyperplasia (1) vs Cancer (2)**				
	1747 cm $^{-1}$	0.0280	−0.0012	0.0056	0.0017
	1689 cm $^{-1}$	0.0836	0.0525	0.0226	0.0029
	1647 cm $^{-1}$	0.2198	0.2030	0.0747	0.0033
	1624 cm $^{-1}$	0.1904	0.1628	0.0650	0.0036
	1531 cm $^{-1}$	0.1946	0.1590	0.0657	0.0021
	1354 cm $^{-1}$	0.0723	0.0081	0.0215	0.0012

© 2020 by the authors. Licensee MDPI, Basel, Switzerland. This article is an open access article distributed under the terms and conditions of the Creative Commons Attribution (CC BY) license (http://creativecommons.org/licenses/by/4.0/).
